# Comparative evaluation of five tacrolimus assays in transplant recipients: implications for optimizing therapeutic drug monitoring

**DOI:** 10.3389/frtra.2025.1716789

**Published:** 2025-12-01

**Authors:** Ivo N. SahBandar, Zhen Zhao, Sabrina E. Racine-Brzostek, Alex J. Rai, Maria Cid, Melissa M. Cushing, Neal Lindeman, Thangamani Muthukumar, He S. Yang

**Affiliations:** 1 Department of Pathology and Laboratory Medicine, NewYork Presbyterian/Weill Cornell Medical Center, New York, NY, United States; 2 Department of Pathology and Laboratory Medicine, Weill Cornell Medical College, New York, NY, United States; 3 Department of Pathology and Cell Biology, Columbia University Irving Medical Center, New York, NY, United States; 4 Department of Transplantation Medicine, NewYork Presbyterian Hospital/Weill Cornell Medical College, New York, NY, United States

**Keywords:** tacrolimus, therapeutic drug monitoring, immunoassay, mass spectrometry, transplant recipients

## Abstract

Tacrolimus is a widely used immunosuppressive therapy in transplant recipients, but its narrow therapeutic index necessitates accurate monitoring. Tacrolimus levels can be quantified using immunoassays (IAs) and liquid chromatography-tandem mass spectrometry (LC-MS/MS), however, differences between these methods may influence clinical decision-making. In this study, we compared two IAs, i.e., chemiluminescent (CMIA) and electrochemiluminescent (ECLIA), with three LC-MS/MS assays in 181 clinical specimens. When compared with the overall mean concentration, all five assays showed strong correlations, though with variability across methods: three LC-MS/MS assays demonstrated correlation coefficients of 0.9927, 0.9612, and 0.9920, while two immunoassays yielded coefficients of 0.9938 and 0.9857. Deming regression analysis revealed slopes of 0.96, 0.94, and 0.93 for the three LC-MS/MS, while the immunoassays showed higher slopes of 1.032 (ECLIA) and 1.21 (CMIA). Bland–Altman analysis indicated systematic underestimation by the LC-MS/MS methods (–7.5%, −18.7%, and −8%) and overestimation by the immunoassays (ECLIA +9.7%, CMIA +18.4%), relative to the overall mean. The two immunoassays showed only moderate agreement with each other (slope = 0.85, intercept = 0.49), and even the LC-MS/MS assays were not fully concordant. Among 47 patients within 3 months post-transplantation and 134 patients beyond 3 months, clinically relevant discrepancies (≥2 ng/ml) between LC-MS/MS and immunoassay results were observed in 13 patients (28%) and 49 patients (37%), respectively. These findings underscore the substantial impact of assay-dependent variability on tacrolimus monitoring and emphasize the need for standardized laboratory practices as well as assay-specific therapeutic ranges to prevent underexposure with rejection or overexposure with toxicity.

## 1 Introduction

Tacrolimus (FK-506) is a calcineurin inhibitor widely used to prevent organ rejection after transplantation by suppressing the host immune responses, specifically by inhibiting the activation and proliferation of CD4+ and CD8+ T lymphocytes (1, 2). In the United States, more than 90% of kidney transplant recipients receive tacrolimus as their primary immunosuppressive therapy (3). Although highly effective, tacrolimus has a narrow therapeutic range. High levels can lead to serious side effects, such as hypertension, neurotoxicity, nephrotoxicity, and increased risks of infection and cancer. Conversely, low levels may result in organ rejection. Therefore, precise therapeutic drug monitoring is critical to maintain safe and effective levels (4–6).

Immunoassays (IA) and liquid-chromatography tandem mass spectrometry (LC-MS/MS) are two major methods for therapeutic drug monitoring of tacrolimus. The two main IA platforms are chemiluminescent immunoassays (CMIA) and electrochemiluminescence immunoassays (ECLIA). On the IA platforms, tacrolimus concentration is quantified by measuring signal intensity proportional to the amount of drug bound to specific antibodies through a chemical reaction or an electrochemical reaction. In contrast, LC-MS/MS employs liquid chromatography to separate tacrolimus from other substances based on its chemical properties, followed by ionization and quantification in a tandem quadrupole mass spectrometer based on its distinct mass-to-charge ratio of the parent compound and its fragments.

Compared with LC-MS/MS, IAs offer a faster turnaround time and simpler operation due to their streamlined workflow, lower technical-skill requirements, and higher throughput. Additionally, IA platforms, such as CMIA and ECLIA, are widely available on fully automated analyzers and have a well-established record of reliable clinical use. Many IA assays are approved by regulatory agencies, including the United States Food and Drug Administration (FDA), and thus, assay validation and implementation are generally straightforward. However, a key limitation of IA is cross-reactivity, where similar compounds, particularly metabolites of the parent drug, can cause significant positive bias and variability in results. This bias may also vary due to manufacturer lot difference, parent co-medications, and hematocrit levels (7–10). In contrast, LC-MS/MS yields highly specific results by directly measuring only the parent drug, making it the gold standard for therapeutic drug monitoring. However, the trade-off is operational complexity, requiring dedicated instrumentations, rigorous sample preparation, and relatively longer turn-around times. In addition, LC-MS/MS testing is classified as a laboratory-developed test and is not FDA-approved, necessitating greater technical expertise for method development and validation. These factors limit its availability compared to IA.

Inter-assay variability among different IAs and between IA and LC-MS/MS can significantly impact tacrolimus dose adjustment and therapeutic monitoring, potentially leading to adverse clinical outcomes (11–15). Studies comparing IA to LC-MS/MS demonstrate that IA measurements are typically 10%–30% higher than those from LC-MS/MS measurements, largely due to IA's cross-reactivity with tacrolimus metabolites (7). This discrepancy is particularly more pronounced in patients with atypical metabolic profiles or those with accumulated metabolites in plasma secondary to renal (16) or hepatic dysfunction (1, 17). Furthermore, switching between assay platforms during routine patient management poses challenges for clinical decision-making for clinical decision-making, as simple mathematical adjustment cannot reliably reconcile difference between the IA and LC-MS/MS values. Currently, no consensus exists on the optimal method for measuring tacrolimus levels.

Previous reports comparing tacrolimus trough levels have primarily been limited to evaluating two assay methods (9–11). In this study, we investigated inter-assay variability in tacrolimus trough concentrations by comparing measurements across two immunoassay methods and three LC-MS/MS-based assays across four independent laboratories, using 181 clinical whole blood specimens collected at a single transplant center. This study aimed to provide a comprehensive evaluation of inter-assay variability to inform tacrolimus therapeutic drug monitoring in transplant recipients.

## 2 Material and methods

A total of 181 whole blood specimens were collected in tubes containing EDTA anticoagulants for routine tacrolimus trough level measurement from transplant patients between April and June 2023 at the NewYork-Presbyterian Hospital/Weill Cornell Medical Center (NYP/WCMC). Each specimen was tested at NYP/WCMC using an in-house IA – the method reported in patient's chart, and LC-MS/MS which was performed offline for comparative analysis. Aliquots of all the specimens were stored at refrigerated temperature and subsequently sent to NewYork-Presbyterian Hospital/Columbia University Irving Medical Center (NYP/CUMC) and two reference laboratories, ARUP laboratories and Quest Diagnostics, to perform the cross-platform comparison. All samples were tested within stability. Weill Cornell Medicine Institutional Review Board (WCM-IRB 19-10020914) approved this study.

Analysis was conducted using two immunoassays: ECLIA (Elecsys, Roche Diagnostics, Indianapolis, IN) used currently at the NYP/WCMC, and the CMIA (ARCHITECT, Abbott, Abbott Park, IL) used currently at NYP/CUMC. The performances of the CMIA and ECLIA methods have been previously described in detail (7). Briefly, the ECLIA tacrolimus assay is a competitive electrochemiluminescence immunoassay performed on pre-treated whole blood. The sample is first mixed with extraction reagent to lyse cells and release tacrolimus, and the supernatant is then analyzed. The CMIA tacrolimus assay is a competitive chemiluminescent microparticle immunoassay which also involves a sample pre-treatment. Both assays provide high throughput and automated workflows. Additionally, three LC-MS/MS assays were used for the sole comparison purpose of this study: LC-MS/MS A (Agilent, Santa Clara, CA) and LC-MS/MS B (Waters, Milford, MA) at two different reference laboratories, i.e., specialized facilities that perform diagnostic testing on specimens received from other laboratories, and LC-MS/MS C (Agilent, Santa Clara, CA) validated at the NYP/WCMC but currently not used for clinical testing. In the LC-MS/MS C method, tacrolimus concentrations in whole blood were determined using an Agilent ultra-high-performance liquid chromatography couple with an Agilent 6470 Triple Quadrupole tandem mass spectrometry. Chromatographic separation was achieved on an InfinityLab Poroshell 120 EC-C18 column (3.0 × 50 mm, 2.7 µm). Whole blood specimens were subjected to protein precipitation with 0.1 M zinc sulfate, followed by centrifugation to obtain the supernatant. Then, the supernatant was loaded onto a solid-phase extraction (SPE) plate for cleanup prior to analysis. Ascomycin serves as the internal standard. Detection was performed in positive electrospray ionization mode using multiple reaction monitoring (MRM), with a precursor ion of m/z 821.5 and two product ions of m/z 768.5 and 84.1 for tacrolimus. The MRN transition for ascomycin was m/z 809.5 -> 756.5. Quantification was based on a six-point calibration curve covering an analytical measurement range of 1.0–40.0 ng/ml. Three levels of quality controls were analyzed before and after patient samples in each batch to ensure assay accuracy and precision.

Comparative analyses were conducted using the average tacrolimus level across all methods as a reference. These included scatter plots, simple linearity regression analysis, Deming regression analysis, Pearson correlation coefficients, Bland-Altman plots, and bias calculations, all performed using GraphPad Prism version 10.1.1. In the Deming regression, constant bias refers to a systematic shift where one method consistently reports higher or lower values across the measurement range (reflected by an intercept different from zero), while proportional bias indicates that the degree of bias changes in proportion to the measured value (reflected by a slope different from one). These assessments help to determine whether discrepancies between methods are fixed across all concentrations or vary with the analyte level.

In clinical practice, tacrolimus dose adjustment is based on clinical judgement. While every transplant patient is unique and providers must consider multiple factors before dose adjustment, based on the practice pattern at our own center (>275 kidney transplants every year, 100% tacrolimus-based protocol), and within the transplant community, we assume that a difference of 1 ng/ml would probably trigger, a difference of 2 ng/ml would almost always trigger, and a difference greater than 3 ng/ml would definitely trigger a dose adjustment. Therefore, the number of cases with tacrolimus level discrepancies (IA vs. LC-MS/MS) of between 1 and 2 ng/ml, between 2 and 3 ng/ml, and greater than 3 ng/ml is presented in both within 3 months post-transplantation and over 3 months post-transplantation phase, respectively.

## 3 Results

### 3.1 Analytic comparisons

A total of 181 patients were included in the study; 63% were male and 37% were female. The median age of the participants was 61 years (interquartile range, IQR 49–69). The most common transplant type was kidney (*n* = 109, 60%), followed by liver (*n* = 41, 23%), heart (*n* = 14, 8%), stem cell (*n* = 11, 6%), simultaneous pancreas and kidney (*n* = 4, 2%), and lung (*n* = 2, 1%). The plurality of patients (*n* = 86, 47%) had more than 12 months elapsed since their most recent transplant, while 48 patients (27%) were between 3- and 12-months post-transplant, and 47 patients (26%) were within 3 months of their most recent transplant. Complete clinical and demographic data are presented in Table 1.

**Table 1:** Demographic and clinical information of the patients in this study.

Demographic and clinical information	*n* = 181
Age, median (IQR)	61 (49–69)
Gender, male, *n* (%)	65 (63)
Transplant type, *n* (%)	
Kidney	109 (60)
Liver	41 (23)
Heart	14 (8)
Stem cell	11 (6)
Simultaneous pancreas and kidney	4 (2)
Lung	2 (1)
Time elapsed from the latest transplantation in months, median (min, max)	12 (1, 361)
≤1 month, *n* (%)	24 (13)
>1 month–3 months, *n* (%)	23 (13)
>3–12 months, *n* (%)	48 (27)
>12 months, *n* (%)	86 (47)

**Figure 1:**
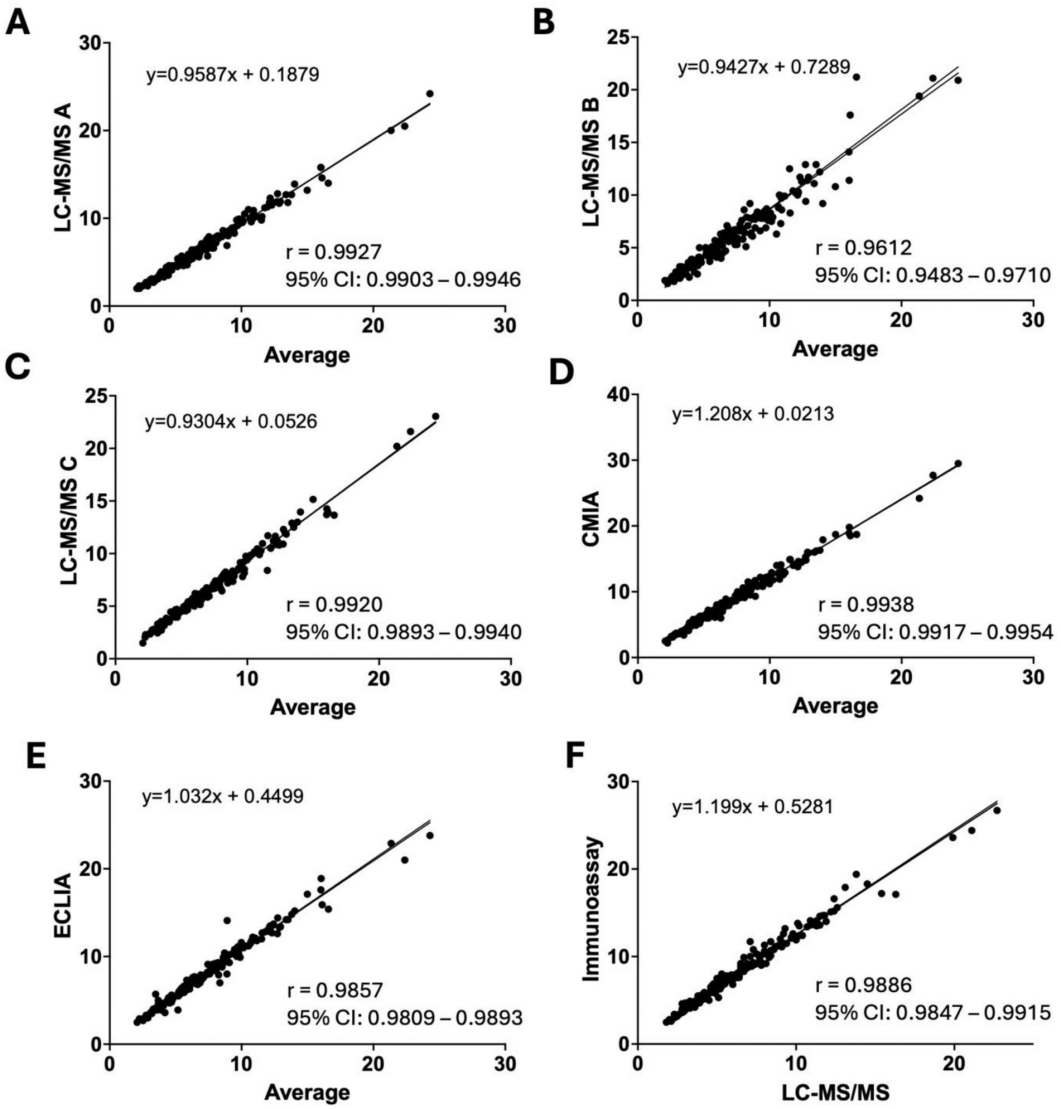
Comparison of tacrolimus levels (ng/ml) measured by LC-MS/MS A **(A)**, LC-MS/MS B **(B)**, LC-MS/MS C **(C)**, CMIA **(D)**, and ECLIA **(E)** against the average values of 5 methods as the reference, and comparison between the average of LC/MS-MS methods vs. average of IA methods **(F)** The two lines represent both Deming and simple linear regression, with the regression equations, regression coefficient, and its 95% confident interval derived from the Deming regression.

Tacrolimus values measured by five different methods were plotted against the average values across all methods (Figures 1A–E), and the average values from the LC-MS/MS assays were plotted against the average values from IA methods (Figure 1F). Deming regression equations were shown in each figure. Although all methods demonstrate a relatively strong linear relationship, notable variations in the slopes and intercepts of the method-specific trendlines indicate systematic discrepancies and biases relative to the average values. The LC-MS/MS methods exhibited a negative bias, whereas the ECLIA and CMIA methods showed a positive bias indicating a tendency to overestimate tacrolimus concentrations compared with LC-MS/MS. The slopes for the three LC-MS/MS methods were 0.96, 0.94, and 0.93, respectively, while the slope for ECLIA was higher at 1.03 and CMIA showed an even greater slope of 1.21, suggesting a more pronounced proportional bias. Correlation coefficients (r) for LC-MS/MS A, B, C, CMIA, and ECLIA against the average were 0.9927, 0.9612, 0.9920, 0.9938, 0.9857, respectively (Supplementary Table S1). Furthermore, the two IA methods had a notable bias with each other, with a slope of 0.85 and an intercept of 0.49 ng/ml (Supplementary Table S1).

**Figure 2:**
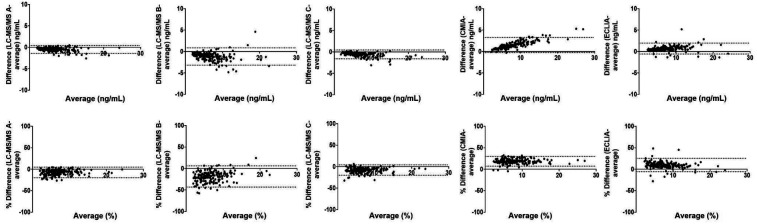
Bland Altman plots. The upper panel shows the absolute levels of tacrolimus (ng/ml), while the lower panel displays the percentage of differences measured by LC-MS/MS A, B, and C, CMIA, and ECLIA, compared to the average values of 5 methods as the reference.

Bland-Altman plot analysis (Figure 2) further revealed that CMIA and ECLIA exhibited positive absolute biases of 1.5 and 0.7 ng/ml, respectively, along with proportional biases of +18.4% and +9.7%, respectively, relative to the average values. On the other hand, LC-MS/MS A, B, and C showed absolute biases of −0.5, −1.2, and −0.6 ng/ml, respectively, and proportional biases of −7.5%, −18.7%, and −8%, respectively.

### 3.2 Clinical impact assessment

**Figure 3:**
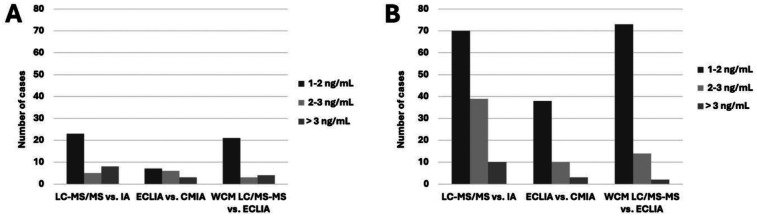
Number of cases with tacrolimus level difference as measured by different methods/platforms. Comparison of tacrolimus levels measured by different methods: average values of 3 LC-MS/MS methods vs. average values of 2 IA methods (left panel), ECLIA vs. CMIA (middle panel), and the LC-MS/MS C vs. ECLIA at the same institution of NYP/WCMC (right panel) in study participants within 3 months **(A)** vs. more than 3 months **(B)** after their latest transplantation.

To evaluate the potential clinical impact of switching between different assay methods while measuring a given patient's tacrolimus levels over multiple runs, we compared the number of cases where tacrolimus levels differed between LC-MS/MS and IA measurements. Because providers strive to maintain tacrolimus trough level within a narrow therapeutic range for a given patient, particularly during the first three months following transplantation, we stratified inter-method discrepancies between IA and LC-MS/MS into multiple categories: 1–2 ng/ml, 2–3 ng/ml, and > 3 ng/ml to assess their potential impact on dose adjustments. Among the 47 patients within three months post-transplantation, 23 (49%) showed a difference of 1–2 ng/ml, 5 (11%) showed a difference of 2–3 ng/ml, and 8 (17%) showed a difference of greater than 3 ng/ml (Figure 3A). Between the two IA methods, the corresponding numbers were 7 (15%), 6 (13%), and 3 (6%), respectively. It is noteworthy that even within the same institution where sample handling conditions were consistent, 5 (11%) samples had a difference of more than 2 ng/ml, highlighting the impact of methodological differences on clinical decision-making. Similarly, in the 134 patients who were >3 months post-transplant, 70 (52%) showed a difference of 1–2 ng/ml; 39 (29%) showed a difference of 2–3 ng/ml; and 10 (8%) showed a difference of greater than 3 ng/ml. Between the two IA methods, the corresponding numbers were 38 (28%), 10 (8%), and 3 (2%), respectively. Importantly, within the same institution, 11% of samples showing a difference of more than 2 ng/ml (Figure 3B).

## 4 Discussion

We investigated the inter-assay variability in tacrolimus trough concentrations across two immunoassay methods and three LC-MS/MS assays conducted at four independent laboratories. To the best of our knowledge, this is the first study to compare multiple IA and LC-MS/MS platforms across both academic and reference laboratories in a large cohort of solid organ transplant recipients. The two IAs showed moderate agreement, while LC-MS/MS assays also exhibited incomplete concordance. Notable biases were identified between IAs and LC-MS/MS platforms, as well as among methods within the same platform type. Importantly, differences exceeding 2 ng/ml were observed in a substantial proportion of patients, particularly between IA and LC-MS/MS methods. These findings underscore significant inter-method variability with critical clinical implications for therapeutic drug monitoring in solid organ transplant recipients, especially during the critical first three months of post-transplantation. Therefore, we recommend that transplant providers maintain consistency in the assay method used for an individual patient and avoid switching between platforms whenever possible.

The ease of measuring tacrolimus trough concentrations and their strong correlation with the area under the curve have established trough level monitoring as a standard laboratory test for transplant recipients. Tacrolimus dosing is primarily guided by trough concentrations. Although early studies evaluating the relationship between trough level and rejection yielded conflicting results, more recent studies with lower target ranges and longer follow-up periods have shown a more consistent association with graft outcomes and adverse effects (19). While high intra-patient variability in tacrolimus levels is a recognized risk factor for adverse outcomes in kidney transplant recipients (20), this variability has typically been attributed to medication non-adherence, genetic factors, or pharmacologic interactions. Assay variability, however, has not been adequately considered in these studies.

Previous studies, including smaller investigations and larger multicenter trials, have compared chemiluminescent microparticle immunoassay (CMIA) and electrochemiluminescence immunoassay (ECLIA) for tacrolimus measurement (7, 21). Inconsistent tacrolimus trough concentrations across different analytical methods have been reported previously (7, 10, 22). However, most prior studies were limited to comparing only two methods, such as LC-MS/MS vs. IA or between two IAs. In contrast, our study comprehensively evaluated five different methods and also examined variations when the analysis was conducted at different laboratories. For measurements by IA, variability in tacrolimus levels may results from differences in assay sensitivity or interference from substances such as biotin, rheumatoid factor, or heterophile antibodies (17, 23, 24). Additionally, discrepancies can arise from cross-reactivity with circulating metabolites, such as 13-O-desmethyl or 31-O-desmethyl tacrolimus, which IAs are known to detect (18), potentially leading to elevated results. Unfortunately, we were unable to identify the presence of these confounding factors in each patient's tacrolimus sample and therefore could not estimate their individual effects on the immunoassay results. On the other hand, LC-MS/MS primarily measures the parent drug, largely excluding metabolites. However, some variation may still arise from sample handling or extraction. Although not perfectly concordant, tacrolimus levels measured by the three LC-MS/MS platforms in this study showed smaller discrepancies than those observed between different IA platforms. The larger discrepancy observed with LC-MS/MS B, compared to LC-MS/MS A and C, may be attributed to instrument differences; specifically, A and C were analyzers from the same vendor, whereas B was from a different manufacturer.

The positive bias observed in IAs relative to the LC-MS/MS raises concerns about potential unintended underdosing of tacrolimus. For instance, an earlier study reported a 26% proportional bias in CMIA relative to the reference method, corresponding to an absolute bias of 2.1 ng/ml (11). Consider a clinical example, a 64-year-old kidney transplant recipient, one year post transplantation, had a tacrolimus trough concentration of 11.7 ng/ml by IA. However, analysis of the same sample by LC-MS/MS yielded a trough level of 7.1 ng/ml. Both measurements met acceptable performance standards and passed quality control checks for their respective platforms. However, an IA result of 11.7 ng/ml might prompt a dose reduction, whereas the LC-MS/MS result of 7.1 ng/ml would indicate no dose adjustment is necessary. Such discrepancies, particularly when patients are monitored across different laboratories or assay platforms, can create uncertainty for clinicians in determining appropriate dose adjustments, especially given the lack of a reliable conversion formula to standardize IA results to LC-MS/MS equivalents (25).

Our findings highlight significant inter-assay variability in tacrolimus trough measurements, underscoring the importance of considering the assay method and laboratory when adjusting doses to minimize adverse effects and allograft rejection. These results reveal a critical need to address variability introduced by different measurement methods and laboratories. To minimize discrepancies, clinicians should avoid switching laboratories or assay methods when monitoring tacrolimus levels in individual patients. Providers must exercise clinical judgment and interpret laboratory results in the context of the patient's overall clinical status to prevent both underexposure and toxicity during tacrolimus therapy. Furthermore, there is an urgent need to identify more sensitive and specific biomarkers for the early detection of transplant rejection and drug-induced toxicity. Improved biomarkers would facilitate timely interventions and optimize immunosuppressive management.

Last but not least, our findings underscore the need for method-specific therapeutic target ranges for tacrolimus. Currently, commonly used therapeutic ranges for tacrolimus are 7–20 ng/ml for kidney transplant recipient within 3 months post-transplantation, and 5–15 ng/ml after 3 months post-transplantation; and 5–20 ng/ml for liver transplant recipients within 12 months post-transplantation. When the current therapeutic ranges were first established, IAs were the predominant platform for drug measurement. A pivotal study by Staatz and Tett helped define these ranges across various organ types and post-transplant timeframes (26). Given the improved performance characteristics of modern assay methods, such as LC-MS/MS, it is both timely and essential to re-evaluate and redefine tacrolimus therapeutic ranges using contemporary platforms.

In conclusion, our findings reveal significant variability in tacrolimus trough concentration measurements across different assay platforms and even among methods within the same platform type, underscoring critical clinical implications for transplant management. These findings aim to deepen transplant providers' understanding of the analytical methods used for tacrolimus measurement, enabling more accurate interpretation of laboratory results and, ultimately, optimizing therapeutic drug monitoring and improving patient outcomes.

## Data Availability

The datasets presented in this article are not readily available because Our IRB does not allow sharing patient data. Requests to access the datasets should be directed to hey9012@med.cornell.edu.
