# Supplementary material for: Comparative evaluation of five tacrolimus assays in transplant recipients: implications for optimizing therapeutic drug monitoring
Source: Front Transplant. 2025 Dec 1;4:1716789. doi: 10.3389/frtra.2025.1716789 (PMC12702874; doi:10.3389/frtra.2025.1716789)

Suppl. Table 1. Deming regression analysis, Pearson correlation coefficient and bias calculation between each and reference method as well as between each method.

|  | **Slope** | **Intercept**  **(ng/mL)** | **r** | **Bias**  **(ng/mL, (%))** |
| --- | --- | --- | --- | --- |
| **Average - A** | **0.9520 (0.9350 – 0.9691)** | **-0.1879 (-0.3712 – -0.045)** | **0.9927** | **-0.50 (-7.51)** |
| **Average - B** | **0.9427 (0.8607 – 1.025)** | **-0.7289 (-1.251 – -0.2067)** | **0.9612** | **-1.16 (-18.67)** |
| **Average - C** | **0.9304 (0.9041 – 0.9567)** | **-0.052 (-0.219 – 0.1135)** | **0.9920** | **-0.57 (-8.0)** |
| **Average - CMIA** | **1.208 (1.180 – 1.236)** | **-0.0213 (-0.205 – 0.163)** | **0.9938** | **1.54 (18.4)** |
| **Average -ECLIA** | **1.032 (0.9827 – 1.082)** | **0.4499 (0.1265 – 0.7734)** | **0.9857** | **0.69 (9.72)** |
| A - B | 0.9843 (0.877-1.094) | -0.5569 (-1.205-0.1030) | 0.9364 | -0.66 (-11.2) |
| A - C | 0.9703 (0.9340-1.007) | 0.1306 (-0.0915-0.3527) | 0.9864 | -0.08 (0.49) |
| A - CMIA | 1.261 (1.218-1.304) | 0.2069 (0.0601-0.4739) | 0.9894 | 2.03 (25.81) |
| A- ECLIA | 1.078 (1.018-1.138) | 0.6448 (0.2707-1.019) | 0.9767 | 1.19 (17.16) |
| B - C | 0.9842 (0.8734-1.095) | 0.6837 (0.1042-0.1236) | 0.9360 | 0.58 (10.71) |
| B - CMIA | 1.296 (1.166-1.426) | 0.8193 (0.1392-1.449) | 0.9414 | 2.69 (36.62) |
| B - ECLIA | 1.10 (0.9671-1.232) | 1.219 (0.5222-1.915) | 0.9264 | 1.85 (28.16) |
| C - CMIA | 1.300 (1.261-1.340) | 0.0323 (-0.2090-0.2736) | 0.9869 | 2.11 (26.28) |
| C - ECLIA | 1.111 (1.042-1.180) | 0.4974 (0.0745-0.9203) | 0.9767 | 1.27 (17.66) |
| CMIA - ECLIA | 0.8517 (0.8028-0.9006) | 0.4943 (0.0974-0.8911) | 0.9743 | -0.84 (-8.70) |

A: LC-MS/MS A, B: LC-MS/MS B, C: LC-MS/MS C, r: correlation coefficient.

Suppl. Figure 1:


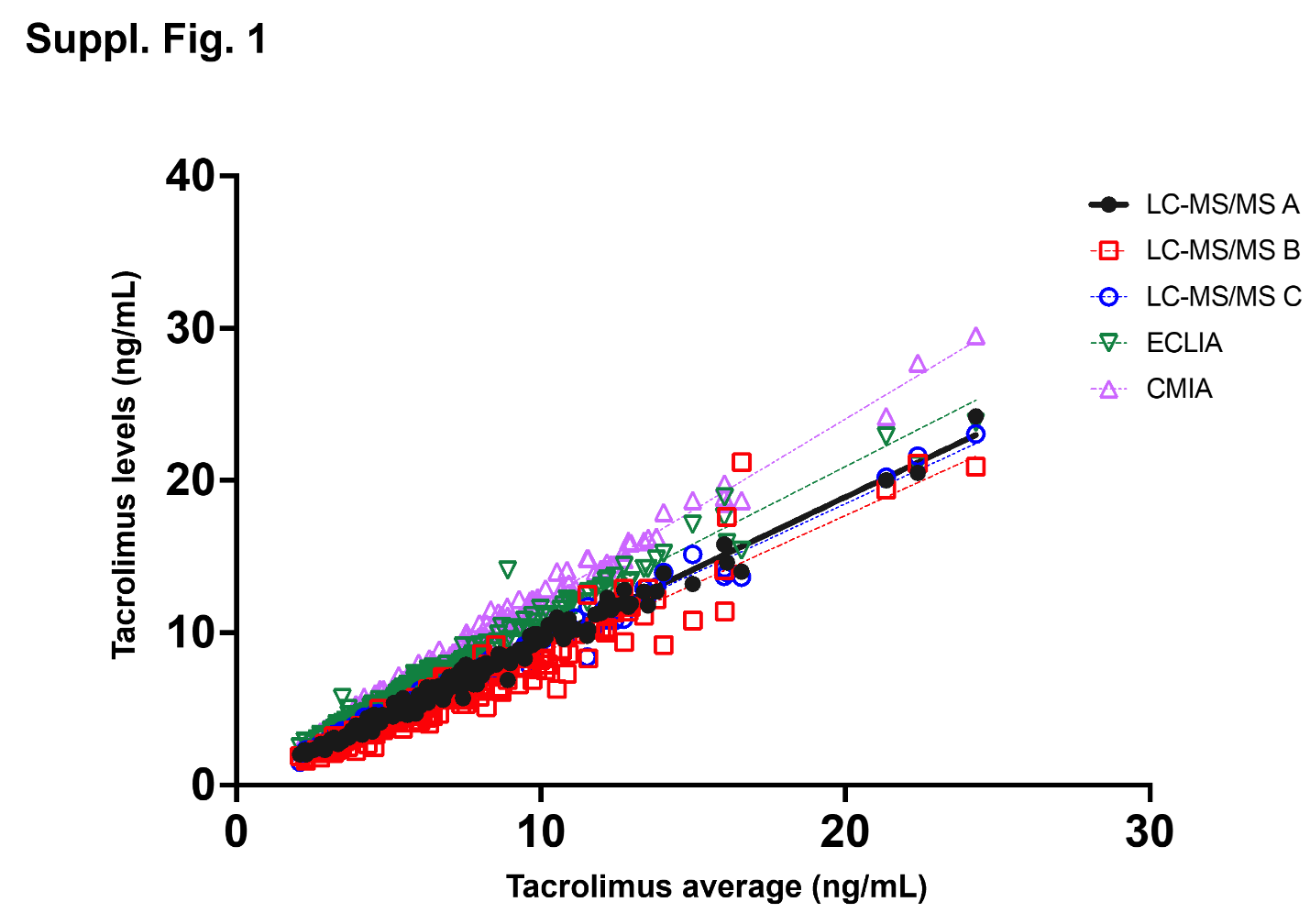

Supplement: Supplementary file 1 [file Datasheet1.docx]
